# 2040. Healthcare-Associated Infection Surveillance During the COVID-19 Pandemic in New Mexico

**DOI:** 10.1093/ofid/ofac492.1662

**Published:** 2022-12-15

**Authors:** Morgan Edwards-Fligner, Erin C Phipps

**Affiliations:** University of New Mexico, Albuquerque, New Mexico; University of New Mexico, Albuquerque, NM; New Mexico Emerging Infections Program, Santa Fe, NM, Albuquerque, New Mexico

## Abstract

**Background:**

The COVID-19 pandemic changed accessibility of care and practices within healthcare environments. This period has been associated with healthcare-associated infection outbreaks and shifts in healthcare-associated infectious disease epidemiology. This study’s objective is to describe changes in rates and characteristics of antimicrobial-resistant gram negative and Clostridioides difficile (CD) infections during the COVID-19 pandemic in Bernalillo County, New Mexico.

**Methods:**

The NM EIP, a collaboration between University of New Mexico and the NM DOH, conducts ongoing laboratory- and population-based surveillance of infectious disease including *Clostridium difficile*, extended-spectrum beta lactamase (ESBL-E) and carbapenemase-producing gram negative bacteria (CRE). Stata statistical software was used for retrospective analysis of rates and characteristics on NM EIP data from Bernalillo county, NM between 2016 and 2021.

**Results:**

Reported *C. difficile* rates decreased from 76 to 49 cases/month and ESBL-producing Enterobacterales decreased from 145 to 86 cases/month during the pandemic period from March-December 2020 compared with the prior 14 months. Monthly case counts for 2020 are lowest during initial public health orders for the state of New Mexico. Rates of CRE remained constant between 2018-2021.

The proportion of CDI cases originating from long-term care facilities decreased significantly from 17.2% to 10.4% (p=0.006) while the proportion attributable to hospital inpatient and community populations remained constant. The proportion of ESBL-E cases from sterile sample sites increased from 3.1% to 4.9% (p=0.05) and the proportion of patients who died within 30 days or prior to discharge increased from 2.2% to 3.2% (p=0.019).

**Figure:**
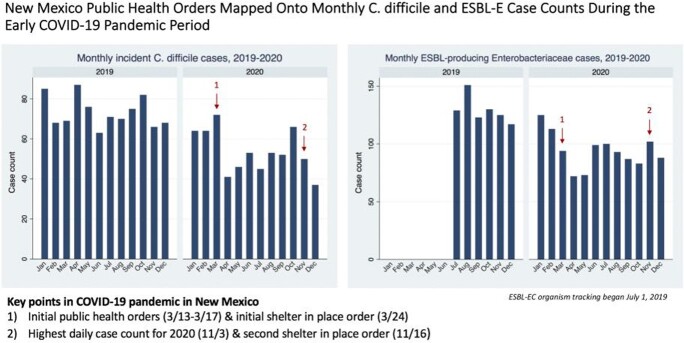

**Conclusion:**

Rates and characteristics of CD and ESBL-E infections in Bernalillo county NM changed significantly during the COVID-19 pandemic, while rates of CRE remained constant. It is still unclear whether this is related to changes in actual disease rates due to risk factor exposure (healthcare), or if this trend reflects changes in care-seeking behavior and/or reporting of cases.

**Disclosures:**

**All Authors**: No reported disclosures.

